# *MTOR*-mutated eosinophilic renal cell carcinomas with loss of chromosome 1 and high metastatic potential: further expanding the spectrum of TSC/mTOR pathway mutated renal tumors

**DOI:** 10.1007/s00428-025-04363-4

**Published:** 2025-11-27

**Authors:** Jung Woo Kwon, Melissa Y. Tjota, Tatjana Antic

**Affiliations:** Department of Pathology, University of Chicago, Chicago, IL USA

**Keywords:** TSC/mTOR pathway, Eosinophilic renal cell carcinoma, Renal tumors

## Abstract

TSC/mTOR pathway alteration is recurrent in numerous renal tumor types, such as eosinophilic vacuolated tumor (EVT), low-grade oncocytic tumor of the kidney (LOT), and eosinophilic solid and cystic (ESC) renal cell carcinoma (RCC). Recently, additional renal tumor types with recurrent TSC/mTOR pathway mutation, such as xanthomatous giant cell RCC and RCC with leiomyomatous stroma, have also been described, further expanding the spectrum of renal tumors with TSC/mTOR pathway mutation. To this date, most renal tumors with TSC/mTOR pathway mutation are reported to be mostly indolent, with only rare cases of metastasis. However, this report describes two cases (one of which was previously published as a case report) of *MTOR*-mutated eosinophilic RCCs with loss of chromosome 1. The tumors mostly exhibit solid/nested and sheet-like growth patterns composed of tumor cells with plump eosinophilic cytoplasm, with occasional vacuolation, pronounced cellular membrane, and occasional perinuclear clearing. The tumor cells showed prominent nucleoli and round nuclei with smooth nuclear membrane. The tumor had occasional binucleated tumor cells with rhabdoid morphology, areas with prominent hyalinized stroma and psammomatous calcification, and tumor cells with blue granules. Immunohistochemistry showed that the tumors were diffusely positive for CD117 and negative for vimentin, and negative for CK7, Melan A, and TFE3. CK20 was positive in both tumors, with diffuse expression in one and focal expression in the other. Next-generation sequencing showed concomitant *MTOR* mutation and loss of chromosome 1. Both tumors displayed metastatic potential. Overall, these *MTOR*-mutated eosinophilic RCCs with loss of chromosome 1 have distinct morphologic, immunohistochemical, and molecular features as well as high metastatic potential, further expanding the spectrum of TSC/mTOR pathway-mutated renal tumors. This tumor requires further investigations due to its high metastatic potential that is infrequently observed in other TSC/mTOR pathway-mutated renal tumors and the potential therapeutic target it may offer in mTOR inhibitor.

## Introduction

Since the clinical adaptation and diagnostic application of various molecular testing techniques, comprehensive research in renal tumors from tuberous sclerosis complex (TSC) syndrome patients has led to the discovery of emerging entities such as eosinophilic vacuolated tumor (EVT) [1–7], low grade oncocytic tumor of the kidney (LOT) [6–13], and eosinophilic solid and cystic (ESC) renal cell carcinoma (RCC) [6, 7, 13–18].

EVT, also called “high-grade oncocytic tumor (HOT)” due to its high-grade cytologic features, is composed of variably vacuolated eosinophilic cells with prominent nucleoli forming predominantly nested architecture in hypocellular or edematous stroma with dispersed single tumor cells and/or small tumor cell clusters very frequently seen. On immunohistochemistry (IHC), EVT is variably positive for CD117 and GATA3, shows scattered positive cells for CK7, and is consistently negative for vimentin [1–7, 19]. LOT is composed of monotonous eosinophilic tumor cells with smooth nuclear contours that form tightly packed tumor nests, sometimes with rare tubules and abrupt stromal edematous change in which small tumor cell clusters and/or single cells appear to float in. On IHC, LOT is consistently positive for CK7 and GATA3 and is consistently negative for CD117 and vimentin [6–13, 19, 20]. ESC RCC is composed of eosinophilic tumor cells forming solid and cystic architecture. On IHC, ESC RCC is usually positive for CK20 and vimentin and is consistently negative for CK7 and CD117 [6, 7, 14–18]. EVT and LOT are thought to behave indolently, with no reported case of metastasis. As for ESC RCC, there are only a few reported cases of lymph node and/or distant metastasis [18, 21–23].

However, these three tumors do not cover the full spectrum of eosinophilic renal tumors with TSC/mTOR pathway alterations. For example, Argani et al. and Xu et al. have described xanthomatous giant cell RCC, another eosinophilic renal tumor with TSC/mTOR pathway alteration which is hypothesized to be in the spectrum of ESC RCC [24, 25].

In 2021, Tjota et al. reported a case of eosinophilic RCC with isolated *MTOR* mutation and loss of chromosome 1 that did not fit into strict diagnostic criteria of EVT, LOT, or ESC RCC by morphology and IHC [26]. This tumor was highly aggressive and metastasized, unlike other eosinophilic renal tumors with TSC/mTOR pathway alteration described in the literature. The tumor stage was pT3aN1 at resection, and the tumor developed metastasis to the liver. In 2023, Caliò also described three RCCs with isolated *MTOR* mutation and loss of chromosome 1 [27]. These tumors also did not fit into strict diagnostic criteria of EVT, LOT, or ESC RCC but interestingly shared striking similarities in terms of morphology, IHC, and clinical behavior with the case reported by Tjota et al., with all three cases developing distant metastasis.

To further characterize this potentially distinct type of RCC with high metastatic potential, this report describes two cases of *MTOR*-mutated eosinophilic RCC with loss of chromosome 1, including the previously reported case by Tjota et al. [26] with an extended IHC panel and additional clinical follow-up.

## Materials and methods

### Study population

Upon retrospective review of the University of Chicago (UChicago) Department of Pathology database between 2005 and 2024, two eosinophilic RCCs with concomitant *MTOR* mutation and loss of chromosome 1 were identified, including the case previously reported by Tjota et al. [26]. Clinical data were obtained through review of electronic medical records.

### Histopathologic analysis and immunohistochemical staining

The hematoxylin and eosin (H&E) and IHC slides were reviewed by two expert genitourinary pathologists. The IHC staining was performed in the clinical laboratory at the University of Chicago using autoimmunostainers (Leica BOND-III & BenchMark XT Ventana) with appropriate controls. The following panel of IHC staining was performed: vimentin (M0725), pankeratin AE1/AE3 (MAB31412), CK7 (M7018), CK20 (M7019), HMB45 (M0634), Melan-A (M7196), PAX8 (10336–1-AP), CD117 (A4502), SDHB (ab14714), TFE3 (354R-16), CD10 (NCL-CD10-270), and GATA3 (390 M-16).

### Next-generation sequencing

A representative formalin-fixed, paraffin-embedded block was selected for NGS in the UChicago Medicine OncoPlus panel, a hybrid-capture panel targeting 1005 cancer-associated genes, of which 168 genes are clinically reported. DNA extraction, DNA quantification, library preparation, and NGS were performed as described [28]. Data analysis was performed using a high-performance computing system (Center for Research Informatics, UChicago) with an in-house developed bioinformatics pipeline. CNV Kit14 software, with additional in-house intrarun normalization and comparison with a pooled cohort of clinical controls, was used to calculate copy number results. The threshold for arm-level copy number changes was an ARM average FC < 0.75 called as deleted and an ARM average FC > 1.5 called as amplified. Somatic variant calls were inspected using Integrated Genomics Viewer (Broad Institute, MIT Harvard, Cambridge, MA). Cosmic and ClinVar were used as additional tools to analyze and confirm detected variants.

## Results

### Clinical features

Patient #1 (previously published by Tjota et al. [26]) was a 65-year-old male who presented with fatigue and low back pain. Workup revealed a left renal mass (8.5 cm in greatest dimension) and small liver and lung nodules suspicious for metastasis. The patient had no other significant medical or family history. The patient subsequently underwent a radical nephrectomy.

Patient #2 was a 55-year-old male who presented with low back pain. Workup revealed a right renal mass (13 cm in greatest dimension). Initial imaging showed no evidence of metastasis. The patient had no other significant medical history or family history. The patient subsequently underwent a radical nephrectomy.

### Histopathology and immunohistochemistry

**Fig. 1:**
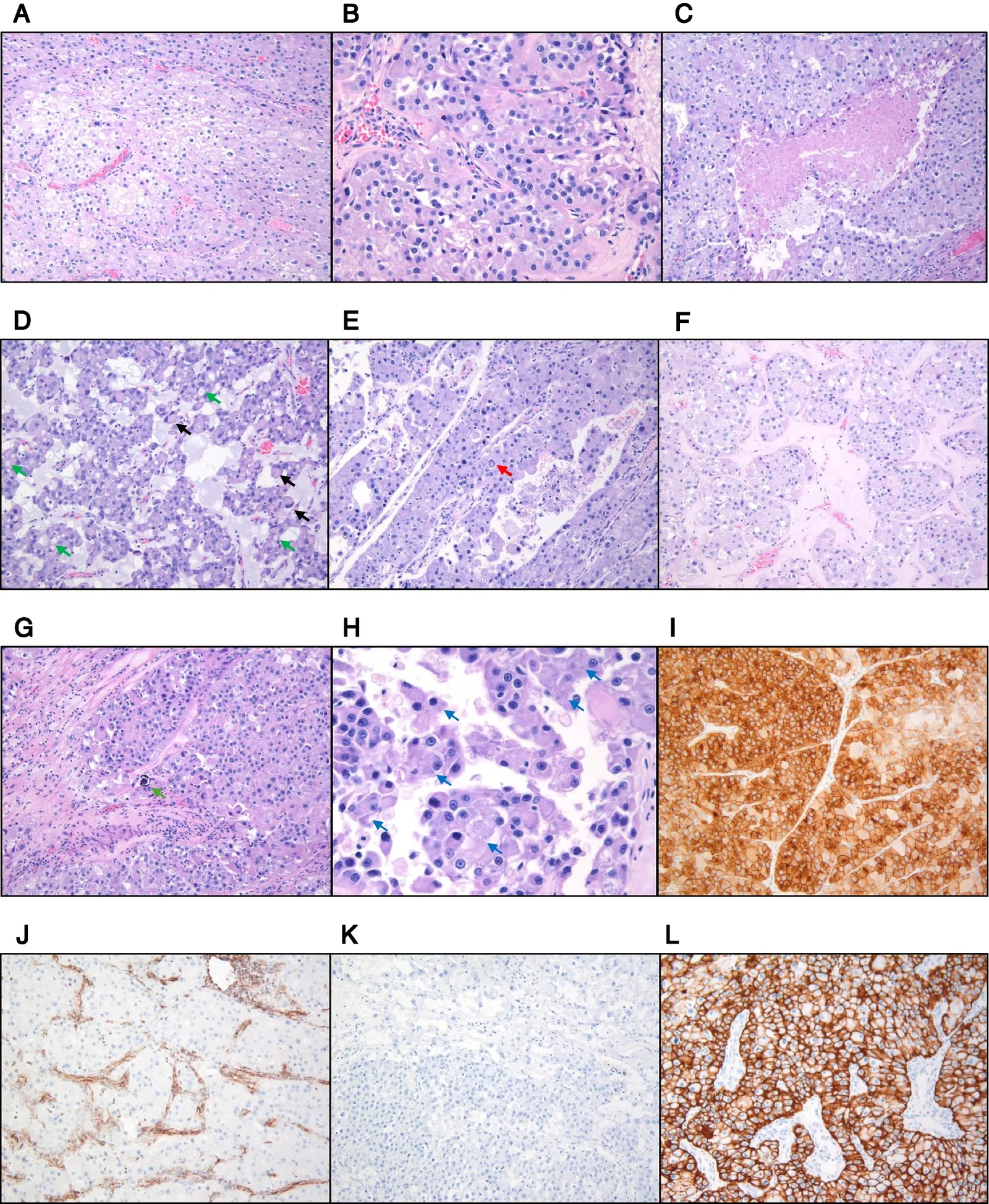
The tumors predominantly exhibited solid/nested and sheet-like architecture composed of eosinophilic tumor cells with pronounced cellular membrane and occasional perinuclear clearing, reminiscent of chromophobe RCC (**A**). However, the tumor cells consistently had round nuclei with smooth nuclear membrane and prominent nucleoli (**A**, **B**). Mitotic figures were frequent (**B**), and tumor necrosis was also present (**C**). The tumors also exhibited other architectural patterns. Some areas showed tumor cells forming loose nests and cords in a background of light-pink amorphous/mucoid material (**D**). In these loosely formed areas with amorphous/mucoid material, binucleated tumor cells with rhabdoid morphology (black arrows; **D**) and vacuolated tumor cells were seen (green arrows; **D**). The tumor cells also occasionally formed pseudotubular and pseudoglandular spaces, with some tumor cells with pseudonuclear inclusions (red arrow; **E**). Prominent hyalinized stroma (**F**) and psammomatous calcification (**G**) were focally seen. Lastly, the tumor cells with “blue granules” reminiscent of ESC RCC were also identified (blue arrows; **H**). By immunohistochemistry, both tumors were positive for PAX8 (diffuse) and CD117 (diffuse; **I**) and negative for vimentin (**J**), similar to chromophobe RCC. However, the tumors were negative for CK7 (**K**). The tumors were both positive for CK20, with tumor #2 showing diffuse CK20 expression (**L**) and tumor #1 showing focal CK20 expression

The two tumors showed similar morphology. The tumors predominantly exhibited solid/nested and sheet-like architecture composed of eosinophilic tumor cells with pronounced cellular membrane and occasional perinuclear clearing, features that are reminiscent of chromophobe RCC (Fig. 1A). However, the tumor cells consistently had round nuclei with smooth nuclear membrane and prominent nucleoli (Fig. 1A, B). Mitotic figures were frequent (Fig. 1B) with up to 20 mitotic figures per 10 high-power fields. Tumor necrosis was also focally (5%) present (Fig. 1C). In addition to solid/nested and sheet-like architecture, the tumors also exhibited other architectural patterns. Some areas showed tumor cells forming loose nests and cords in a background of light-pink amorphous/mucoid material (Fig. 1D). In these loosely formed areas with amorphous/mucoid material, binucleated tumor cells with rhabdoid morphology (black arrows; Fig. 1D) and vacuolated tumor cells were seen (green arrows; Fig. 1D). The tumor cells also occasionally formed pseudotubular and pseudoglandular spaces, with some tumor cells having pseudonuclear inclusions (red arrow; Fig. 1E). Prominent hyalinized stroma (Fig. 1F) and psammomatous calcification (Fig. 1G) were focally seen. Lastly, the tumor cells with “blue granules” reminiscent of ESC RCC were also identified (blue arrows; Fig. 1H).

The IHC features of the tumors are summarized in Table 1. Both tumors were positive for PAX8 (diffuse, > 90%) and CD117 (diffuse, > 90%; Fig. 1I) and negative for vimentin (Fig. 1J), similar to chromophobe RCC. However, the tumors were negative for CK7 (Fig. 1K). Beyond CK7, both tumors demonstrated interesting keratin staining. For CK20, Tumor #2 showed diffuse (> 90%) CK20 expression (Fig. Fig. 1L), while Tumor #1 showed focal (5–10%) CK20 expression. Both tumors were negative for pan-keratin AE1/AE3. Additionally, both tumors were negative for Melan A, TFE3, and CD10 and had retained SDHB expressions.

**Table 1:** Reported cases of MTOR-mutated RCC with concomitant loss of chromosome 1 in literature

Case	Age/sex	Size (cm)	Stage	PAX8	CD117	CK7	CK20	AE1/AE3	Melan A	HMB45	Vimentin	*MTOR* mutation	Chr 1	Follow up (months)
1 (UChicago)	63/M	8.5	pT3aN1	+	+	-	+ (focal)	-	-	N/A	-	c.7279_7280delinsAA (p.L2427K)	Loss	Liver metastasis, 153 months alive
2 (UChicago)	55/M	12	pT3aNx	+	+	-	+	-	-	N/A	-	c.4348_4368del (p.Y1450_W1456del)	Loss	Right rib metastasis and paracaval lymphadenopathy, 16 months alive
3 (Caliò et al.)	21/F	9.5	pT2aN1	+	+ (focal)	-	+ (5%)	-	-	-	-	c.4348_4368del (p.Y1450_W1456del)	Loss	Lymph node metastasis, 30 months alive
4 (Caliò et al.)	58/F	6.5	pT1bNx	+	+ (focal)	-	+ (40%*)	-	-	-	-	c.7280 T > A (p.L2427Q)	Loss	Skull metastasis after 132 months, 135 months alive
5 (Caliò et al.)	49/M	3.6	pT1aNx	+	+ (focal)	-	-	-	-	-	-	c.7280 T > A (p.L2427Q)	Loss	Liver metastasis after 36 months, 64 months alive

*Only positive in the skull metastasis

### Next-generation sequencing

Tumor #1 exhibited a pathogenic mutation in *MTOR* (c.7279_7280delinsAA, p.L2427K; VAF: 57%) and a loss of chromosome 1 in copy number variant analysis as previously published [26]. Apart from the loss of chromosome 1, no significant copy number changes were observed. Tumor mutational burden was 3.0 mutations per megabase. The tumor was microsatellite stable (MSS). The patient reportedly underwent germline testing in 2022 by Invitae multi-gene panel (https://www.invitae.com/us/providers/test-catalog), which revealed no pathogenic variant in all genes tested, but a variant of uncertain significance in *BRCA1* (heterozygous; c.3518G > A; p.Ser1173Asn) was seen.

Tumor #2 also exhibited a pathogenic mutation in *MTOR* (c.4348_4368del, p.Y1450_W1456del; VAF: 27%) as well as a loss of chromosome 1 in copy number variant analysis. Apart from the loss of chromosome 1, no significant copy number changes were observed. Tumor mutational burden was 1.2 mutations per megabase. The tumor was microsatellite stable (MSS).

### Clinical follow-up

Tumor #1 was staged as pT3aN1 due to biopsy-confirmed metastasis in a retroperitoneal lymph node. Following surgery, the patient was first treated with sunitinib with a stable disease, followed by nivolumab, which led to a partial response. As liver nodules were persistent and showed slight enlargement, a targeted biopsy was performed and confirmed metastatic renal cell carcinoma. Axitinib was then started, followed by hepatic arterial yttrium-90 microspheres for persistent liver-only disease. The patient also received radiation for increasing periportal abdominal lymphadenopathy in 2022. Since then, the patient has been alive with a stable disease 153 months after the initial diagnosis.

Tumor #2 was staged as pT3aNx as it showed invasion into renal sinus soft tissue and segmental branch of renal vein as tumor thrombi. On the first surveillance CT Chest–Abdomen–Pelvis (CAP) 3 months after the surgery, an enlarged paracaval lymph node measuring 1.6 × 1 cm and a 16 mm right rib lesion were identified. A follow-up scan 4 months after the initial surveillance CT CAP showed increased size of retrocaval adenopathy (1.6 × 1 cm → 3.3 × 1.9 cm) and a growth of the right rib lesion (16 mm → 21 mm), consistent with clinicoradiologic recurrence/progression of the disease. While the systemic therapy is held off until further disease progression with greater tumor burden, the patient received localized radiation therapy to the right rib lesion due to pain associated with the lesion. The patient is currently alive with stable disease 16 months after the initial diagnosis.

## Discussion

TSC/mTOR pathway alteration has been described in numerous renal tumors, including EVT, LOT, and ESC RCC. ESC RCC is listed in the 5th edition of WHO Classification of Tumours—Urinary and Male Genital Tumours as a distinct recognized entity, while EVT and LOT are listed as emerging entities under “Other oncocytic tumours of the kidney” section. Beyond these three, TSC/mTOR pathway alteration has also been described in xanthomatous giant cell RCC [24, 25] and RCC with leiomyomatous/fibromyomatous stroma [29–33]. Additionally, TSC/mTOR pathway alterations can also manifest as secondary alterations in known RCC subtypes, such as clear cell renal cell carcinoma with secondary *MTOR* mutation [34, 35]. While various renal tumors with TSC/mTOR pathway alteration have been described, all these tumors with TSC/mTOR pathway alteration were mostly thought to be quite indolent in terms of clinical behavior, with only rare cases so far reported to display metastatic potential [18, 21, 22].

The current study reports two *MTOR*-mutated eosinophilic RCCs with loss of chromosome 1 that display high metastatic potential, which further expands the spectrum of TSC/mTOR pathway mutated renal tumors. The tumors in the current study are highly similar to the three aggressive *MTOR*-mutated RCCs with concomitant loss of chromosome that were recently described by Caliò et al. [27] (Table 1). Morphologically, these tumors are composed of eosinophilic cells with smooth round nuclei and prominent nucleoli that predominantly form solid and nested architecture. Additionally, the occasional large, atypical tumor cells with rhabdoid appearance and basophilic granules were seen. IHC showed that these tumors are consistently positive for CD117 (5/5) and consistently negative for vimentin (0/5), CK7 (0/5), pankeratin AE1/AE3 (0/5), and Melan A (0/5). The majority of the tumors were also variably positive for CK20 (4/5), displaying focal to diffuse expression. Molecularly, all the tumors showed *MTOR* mutation with concomitant loss of chromosome 1, with three of the five cases showing *MTOR* alteration in exon 53 (p.L2427Q in two cases and p.L2427K in one case) and the remaining two cases showing the *MTOR* alteration in exon 30 (p.Y1450_W1456del in both cases). All five cases developed metastatic disease to lymph nodes and/or distant sites.

Collectively, *MTOR*-mutated eosinophilic RCCs with loss of chromosome 1 exhibit distinct morphologic and immunohistochemical features and high metastatic potential that are not usually observed in other TSC/mTOR pathway altered renal tumors. Further investigating and defining this unique tumor type for pathologists and clinicians altogether may be of clinical significance due to this tumor’s mimicry of other eosinophilic renal tumors as well as potential adverse clinical consequences when this unique tumor is not recognized. Just to provide some specific examples of its potential mimicry of other eosinophilic renal tumors, due to the areas with perinuclear halos and the immunohistochemical profile (CD117 +/vimentin-), this *MTOR*-mutated eosinophilic RCC with loss of chromosome 1 can mimic chromophobe RCC. This tumor can also mimic ESC, as this tumor can have tumor cells with basophilic granules and display CK20 positivity. Neither of these tumors is generally considered to be RCC subtypes with high metastatic potential, and misdiagnosis of these *MTOR*-mutated eosinophilic RCC with loss of chromosome 1 as chromophobe RCC or ESC could potentially lead to a missed window of opportunity for treatment as the patients may be undergoing less rigorous surveillance and only come back to the hospital when the disease is already widespread. Additionally, when misdiagnosed as chromophobe RCC, the patient may be placed under a less-than-optimal treatment regimen and lose out on a potential therapeutic target in *MTOR* mutation. In fact, one of the patients described in the series by Caliò et al. received Everolimus (mTOR inhibitor) as a second-line therapy for the metastatic disease that showed progression despite first-line therapy and showed clinical response to the mTOR inhibitor. The patients in the current series responded well to first-line therapies given and have not needed a second-line therapy, for which mTOR inhibitor is being considered an option if the diseases progress.

To summarize, *MTOR*-mutated eosinophilic RCC with loss of chromosome 1 described in this study further expands the spectrum of renal tumors with TSC/mTOR pathway mutations. For surgical pathologists, it may be of clinical significance to recognize these RCCs from other eosinophilic renal tumors due to their aggressive clinical behavior unlike other TSC/mTOR pathway altered renal tumors. The accurate distinction of this RCC subtype apart from other eosinophilic renal tumors could subsequently have potential clinical significance in the patient follow-up, surveillance, and therapeutic targets it may offer.
